# Evaluation of Isokinetic Knee Strengths after ACL Reconstruction with Quadrupled Semitendinosus Suspensory Femoral and Tibial Fixation versus Four-Strand Semitendinosus and Gracilis Suspensory Femoral and Tibial Screw Fixation

**DOI:** 10.3390/jcm12124004

**Published:** 2023-06-12

**Authors:** Mehmet Halis Cerci, Ali Kerim Yilmaz, Lokman Kehribar, Serkan Surucu, Mahmud Aydin, Mahir Mahirogullari

**Affiliations:** 1 School of Medicine, Istanbul Arel University, Istanbul 34537, Turkey; 2 Departments of Recreation, Faculty of Yaşar Doğu Sport Sciences, Ondokuz Mayıs University, Samsun 55100, Turkey; 3 Department of Orthopedics and Traumatology, Samsun University, Samsun 55090, Turkey; 4 Department of Orthopedics and Traumatology, Yale University School of Medicine, New Haven, CT 06510, USA; 5 Orthopedics and Traumatology, Haseki Education Research Hospital, Istanbul 34096, Turkey; 6 Orthopedics and Traumatology, Sisli Memorial Hospital, Istanbul 34384, Turkey

**Keywords:** ACL reconstruction, cortical suspensory fixation, interference screw, isokinetic strength

## Abstract

Introduction: The purpose of this study was to demonstrate that patients undergoing ACL reconstruction with quadrupled semitendinosus suspensory femoral and tibial fixation have comparable results in muscle strength and knee function to those undergoing ACL reconstruction with four-strand semitendinosus-gracilis suspensory femoral fixation and a bioabsorbable tibial interference screw fixation. Materials and Methods: Between 2017 and 2019, 64 patients who were operated on by the same surgeon were included. Patients underwent ACL reconstruction technique with quadrupled semitendinosus suspensory femoral and tibial button fixation in Group 1, and patients underwent ACL reconstruction with coupled four-strand semitendinosus-gracilis suspensory femoral fixation and a bioabsorbable tibial interference screw in Group 2. Evaluation of patients was performed with the Lysholm and Tegner activity scale preoperatively and at the 1st and 6th months postoperatively. At the 6-month visit, isokinetic testing of the operated and non-operated limbs was performed in both groups. Results: There was no significant difference in the age, weight, and BMI values of the patients in Groups 1 and 2 (*p* < 0.05). According to the strength values of the operated sides of the patients in Group 1 and Group 2, there was no significant difference in the angular velocities of 60° s^−1^, 180° s^−1,^ and 240° s^−1^ in both extension and flexion phases between the operated sides of Groups 1 and 2 (*p* < 0.05). Conclusions: Patients who have ACL reconstruction with quadrupled semitendinosus suspensory femoral and tibial fixation have comparable muscle strength and knee function to those who undergo ACL reconstruction with four-strand semitendinosus-gracilis suspensory femoral fixation and a bioabsorbable tibial interference screw.

## 1. Introduction

The knee joint is the most often injured during recreational activities, with some studies stating that anterior cruciate ligament (ACL) injuries account for 50% of all injuries [1]. The incidence of ACL injuries in the general population is estimated to be between 30 to 78 per 100,000 people [2]. Recently, the most often utilized approach for managing complete ACL injuries has been arthroscopic-assisted ACL reconstruction, which involves the replacement of the torn ligament with an autograft or allograft [3]. With the development of treatment options for anterior cruciate ligament injury, the number of studies comparing treatment techniques has increased [4,5,6]. Some of these studies compare the strength of the isometric quadriceps and hamstring muscles [7]. Coupling hamstring tendons for anterior cruciate ligament reconstruction has been demonstrated to result in decreased hamstring muscle strength at high knee flexion angles [8,9]. Additionally, it has been demonstrated that hamstring tendon removal results in a loss of inner tibial rotation [10]. However, no clinical research has yet proved that one graft is preferable to another in terms of the reduction in muscular strength output that results [11].

Strength deficits, muscle imbalances, and quadriceps inhibition are common after the reconstruction of ACL [10,11]. Isometric and isokinetic strength tests have been recognized as valuable tools for evaluating the rehabilitation process and determining whether an individual should return to sports (RTS) [12]. When returning to strenuous activities, current literature suggests a side-to-side difference of less than 10–15% in muscle strength as acceptable for ACL tear patients [13]. At the same time, the assessment of quadriceps and hamstring strength in RTS settings is commonly done using an isokinetic dynamometer [14,15]. Typically, asymmetries between the operated and non-operated leg as well as the hamstring/quadriceps (H/Q) ratio, are calculated using maximum torque values [16,17]. Several studies showed decreased strength levels from pre-surgery to 6 months postoperatively, with a subsequent increase in strength during the later stages of rehabilitation [18].

The purpose of this study was to demonstrate that patients undergoing ACL reconstruction with quadrupled semitendinosus tendon suspensory femoral-tibial button fixation have comparable results in muscle strength and knee function to those undergoing ACL reconstruction with four-strand semitendinosus-gracilis tendons suspensory femoral fixation and a bioabsorbable tibial interference screw fixation. This study hypothesized that a single hamstring tendon harvesting technique would have a less detrimental impact on knee flexion than two hamstring tendon harvesting techniques.

## 2. Material and Methods

This retrospective study was conducted in accordance with the ethical standards of the local ethics committee (Protocol No: GOKA/2021/17/14). The informed consent form was taken from the participants of this study.

**Figure 1:**
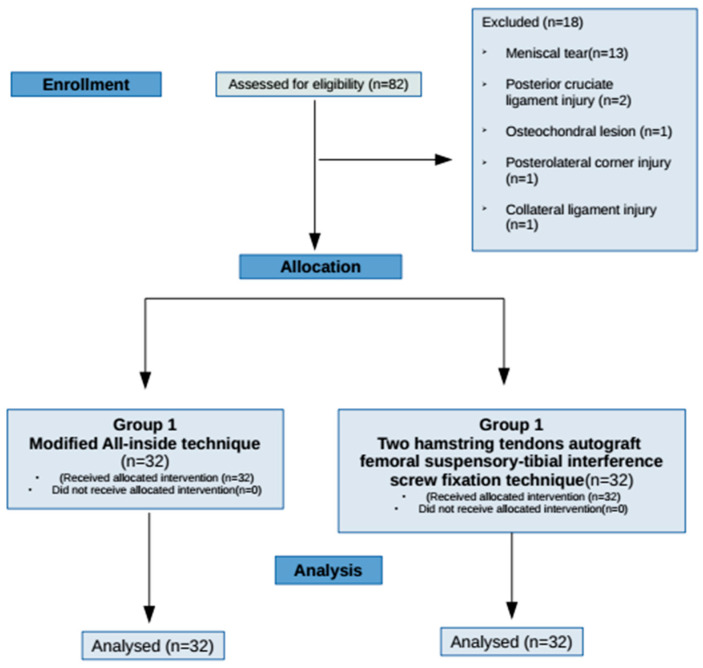
The flow chart of recruitment.

Patients who underwent ACL reconstruction with quadrupled semitendinosus tendon suspensory femoral-tibial button fixation and with four-strand semitendinosus-gracilis tendons suspensory femoral fixation and a bioabsorbable tibial interference screw fixation technique between 2017 and 2019 were evaluated. The inclusion criteria were an isolated anterior cruciate ligament rupture in 1 knee without concurrent meniscal, chondral, or other ligamentous injuries, absence of other neuromuscular or musculoskeletal diseases, and absence of contralateral knee injury or surgery history. Patients who had not completed the rehabilitation and follow-up protocol were excluded. Following the exclusion criteria, subsequently, 32 subjects were assigned to each group, and a total of 64 patients’ data were studied retrospectively (Figure 1).

### 2.1. Surgical Technique

#### 2.1.1. Group 1: Only the Semitendinosus Tendon Autograft Tibial-Femoral Suspensory Fixation Technique (Modified All-Inside Technique)

In this technique, only the semitendinosus tendon was harvested with a tendon stripper and prepared as a tendon autograft. It was prepared as in the Graftlink technique, and quadrupled autograft was finally put out. Both ends were secured with a Femobutton suspension device (Orthomed, Turkey). Graft diameters were between 8–10 mm, and length was always kept between 65–70 mm to make the fixation system work. The location of the femoral tunnel was set with the femoral aimer, which was introduced from an anteromedial portal, and the location of the tibial tunnel was set with a 60–65° adjusted tibial aimer which was also introduced from the anteromedial portal. Both tunnels were drilled in an antegrade fashion, and the diameters were adjusted according to autograft thickness. Autografts were fixed femoral and tibial tunnels with suspensory fixation device combinations [19].

#### 2.1.2. Group 2: Semitendinosus and Gracilis Tendons Autograft Femoral Suspensory-Tibial Interference Screw Fixation Technique

In this technique, 2 hamstrings, the semitendinosus and gracilis tendons, were harvested with a tendon stripper and prepared as tendon autograft in two folded fashion (four strands). Femoral and tibial tunnels were drilled as in the Modified All-Inside Technique. Autograft was fixed to the femoral tunnel with a button suspensory device (Endobutton CL, Smith and Nephew or Femobutton, Orthomed, Turkey) and tibial tunnel with a bio-interference screw (Biosure H, Smith and Nephew).

Patients’ age, BMI, and pre-operational Lysholm and Tegner scores were considered as criteria to match patients. Evaluation of patients was performed with the Lysholm and Tegner activity scale preoperatively and at the 1st and 6th months postoperatively. After surgery, all the patients were included in the same rehabilitation program. At the 6-month visit, isokinetic testing of the operated and non-operated limbs was performed in both groups.

### 2.2. Rehabilitation Protocol

The postoperative rehabilitation protocol was applied similarly to both groups by the same physiotherapist. A rehabilitation protocol which was developed according to the literature and constituted of 4 phases, was applied for each case [20,21].

Phase 1 was begun the day after surgery and continued for 4 weeks. It consisted of terminal isometric quadriceps contraction, patellar mobilization, straight leg raising (SLR), neuromuscular electrical stimulation (NMES), active ankle range of motion exercises in all directions, passive knee flexion as pain allowed and, in 3–4 weeks (90 degree knee flexion and complete knee extension were aimed for), mobilization with a couple of crutches without giving weight to operated side in the first week after surgery.

Phase 2 was between the 4th and 6th weeks after surgery. Isometric quadriceps contraction and patellar mobilization exercises were continued as in Phase 1. In addition to this isotonic quadriceps exercise, vastus medialis obliques, gluteus medius-maximus and hamstring strengthening exercises were performed. While in this phase, walking was improved gradually, and full weight bearing was finally aimed for. Closed kinetic chain exercises were preferred after controlled weight bearing was achieved. Isotonic SLR in all directions, lateral step-ups, and mini squats were performed, and balancing and proprioceptive training were added. If needed continuous passive motion (CPM) machine was used. One hundred thirty-five degrees of knee flexion was aimed at the end of this phase.

Phase 3, returning to daily activity, took place between the 6th and 10th weeks. Fast walking, slow-fast straight-line running, climbing stairs up and down, balancing and elastic-band resistance exercises were performed.

Phase 4, returning to full activity, took place between the 10th and 12th weeks. The intensity of the resistance exercises was increased. Progressive resistance exercises (PRE) and endurance, agility, and plyometric exercises were begun.

### 2.3. Experimental Design

In this study, a 3-blind quasi-experimental design was performed. The technician who was responsible for knee strength measurement and the analyst were blinded to the surgical technique. The patients were blinded regarding autograft type and surgical technique. Isokinetic knee strengths were measured, which are used in the determination of knee strength with knee extension (Ex) and flexion (Flx). The patients were informed during the initial visit about the test protocols to be implemented and the measurement of height, weight, and body mass index (BMI). Isokinetic knee strength tests for concentric/concentric (Con/Con) contractions (60° s^−1^, 180° s^−1^, and 240° s^−1^) at the defined angular velocities were assessed in the following visit. All different angular velocities are isokinetic knee strength tests performed on the operated knee and the non-operated knee. Patients were informed to avoid any physical activity or exercise before testing.

### 2.4. Isokinetic Knee Strength Measurements

A computer-controlled isokinetic dynamometer (Humac Norm Testing and Rehabilitation System, CSMI, Stoughton, MA, USA) was used to assess the patients’ isokinetic test of concentric hamstrings and quadriceps strength. The height and weight measurements were performed by using a SECA BMI scale (Medical Measuring Systems and Scales, Hamburg, Germany) just prior to the isokinetic knee strength measurement. To measure their height, all patients were asked to stand with heels, buttocks, and upper back against a stadiometer. The subjects were asked to distribute their body weight across both feet equally while keeping their shoulders relaxed, legs straight, and arms at their sides while their weight was measured. The protocol started with cycling at 100–120 W with a cadence of 60–70 cycles per minute for 8 min, then the seat and dynamometer were adjusted according to the fixed protocol set for knee Ex and Flx strengths [22]. The range of motion (ROM) of the patient’s knee joint was brought to a position of 0–90° according to this procedure. The back support of the seat was adjusted at a hip joint angle of 85°. At the level of the lateral femoral epicondyle, the rotation of the dynamometer arm was defined. For lower leg attachment, the pad was placed proximal to the lateral malleolus. The trunk was stabilized with chest and pelvic belts while keeping arms crossed and hands on the contralateral shoulder. The contralateral ankle was placed on the leg stabilizer to avoid the movement of the contralateral limb. The rotational axis of the knee joint and rotational axes were measured on the same line before all tests. Before the measurements, the torque value of the knee generated by the leg in a free position at 90° Ex was measured by a dynamometer in all patients to remove the influence of gravity. Before beginning the test, to realize the best strength performance for subjects, all the patients were required to apply the strength of the knee to a maximum level to provide a positive test and to achieve maximum results.

The knee isokinetic strength measurements of the patients were performed for Con/Con contractions at angular velocities of 60° s^−1^ (rest of 15 s after 4 repeated trials and 5 repeated main tests), 180° s^−1^ (rest of 15 s after 4 repeated trials and 5 repeated main test) and 240° s^−1^ (rest of 15 s after 4 repeated trials and 15 repeated main test) found in the fixed protocol of the dynamometer. Measurements were begun with the operated knee first. The patients were given 30 s rest intervals during the changeover between angular velocities. Verbal reminders about the simple push/pull and the number of repeats remaining were given to all patients throughout the measurements. To increase the peak torque (PT) values of patients to the highest level, motivating phrases were continuously spoken at a high frequency. The PT values obtained from the tests were recorded in Newton meters (Nm).

A few subjects could not complete the isokinetic knee strength measurements properly. For these subjects, measurements were repeated at least 8 days later.

## 3. Statistical Analysis

The power of the present study was determined by using G*Power 3.1.9.4 (Universität Düsseldorf, Germany) [23]. A study that had achieved a power of 0.99 with a 0.25 effect size and an α of (*p*) 0.05 was taken as a reference [24]. Thirty-two subjects in each group were determined as enough to detect a statistically significant difference between the groups while postulating a *p*-value of 0.05 and a 1-β value of 0.80.

The data were statistically evaluated using the SPSS 22.0 package application. The Kolmogorov–Smirnov test was utilized to examine the normality assumption of the data, and it was established that the data exhibited a normal distribution. A homogeneity test was made with Levene’s test and found that the variance was homogeneous. We used a paired sample *t*-test for operated and non-operated knees and an independent sample *t*-test for Group 1 and Group 2. The effect sizes based on Cohen’s d [(M2 − M1)⁄SDpooled] were determined. The statistical results were evaluated within a 95% confidence interval and at a significance level of *p* < 0.05.

## 4. Results

Thirty-two of the 64 patients were operated on with a quadrupled semitendinosus tendon suspensory femoral-tibial button fixation, and 32 patients were operated on with four-strand semitendinosus-gracilis tendons suspensory femoral fixation and a bioabsorbable tibial interference screw fixation technique. Patients who operated with quadrupled semitendinosus tendon suspensory femoral-tibial button fixation technique were classified as Group 1, and patients who operated with the classical method as Group 2. All of the patients were male. The mean age in Group 1 was 26.15, and in Group 2 was 22.65 years. The average BMI values were 24.45 in Group 1 and 24.05 in Group 2 (Table 1). When Table 1 was analyzed, no significant difference was found in the age, weight, and BMI indexes of the patients in Groups 1 and 2 (*p* < 0.05).

The strength values of the operated and non-operated sides of Group 1 and Group 2 patients were compared. According to these results, a statistically significant difference was found in favor of the non-operated side in the angular velocities of 60° s^−1^, 180° s^−1^ and 240° s^−1^ in the extension phase and 180° s^−1^ and 240° s^−1^ angular velocities in the flexion phase of the patients in Group 1 (*p* < 0.05). The strength values of the patients in Group 2 were statistically significant in favor of the non-operated side in the angular velocities of 60° s^−1^ and 240° s^−1^ in the extension phase and 60° s^−1^ and 240° s^−1^ angular velocities in the flexion phase (*p* < 0.05; Table 2).

The strength values of the non-operated sides of the patients in Group 1 and Group 2 were compared between the groups. When the strength values of the non-operated sides of the patients in Group 1 and Group 2 were compared, a significance in favor of Group 1 was found in the angular velocities of 60° s^−1^, 180° s^−1,^ and 240° s^−1^ in both extension and flexion phase (*p* < 0.05). The results show that patients in Group 1 revealed higher strength values (Table 3).

The strength values of the operated sides of the patients in Group 1 and Group 2 were compared with covariance analysis. According to these results, there was no significant difference in the angular velocities of 60° s^−1^, 180° s^−1,^ and 240° s^−1^ in both extension and flexion phases between the operated sides of Groups 1 and 2 (*p* < 0.05; Table 4).

**Figure 2:**
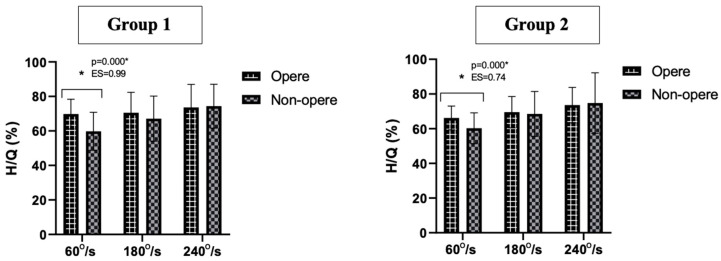
Comparison of the H/Q ratios of operated and non-operated sides in Groups 1 and 2 patients. * *p* < 0.001.

The hamstring/quadriceps (H/Q) ratios of the operated and non-operated sides of the patients in Group 1 and Group 2 were compared. According to these results, it was determined that the patients in Group 1 had higher H/Q ratios on the operated side compared to the non-operated side at angular velocities at 60° s^−1^ (*p* < 0.05). In Group 2 patients, statistical significance was also found at an angular velocity of 60° s^−1^ (*p* < 0.05; Figure 2).

**Figure 3:**
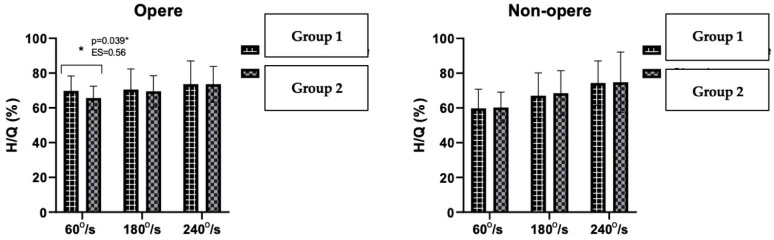
Comparison of the H/Q ratios of Groups 1 and 2 in operated and non-operated sides. * *p* < 0.005.

The H/Q ratios of the operated and non-operated sides of the patients in Group 1 and Group 2 were compared between the groups. According to these results, statistical significance was found in the operated sides at an angular velocity of 60° s^−1^ (*p* < 0.05). In the non-operated sides of the patients, there was no statistically significant difference between Groups 1 and 2 (Figure 3).

## 5. Discussions

In this study, the dynamometric values of patients who had ACL reconstruction with both hamstring tendon suspensory femoral fixation and a bioabsorbable tibial interference screw fixation versus the single hamstring tendon reconstruction technique suspensory femoral-tibial button fixation were evaluated.

It was hypothesized that a single tendon harvesting technique would cause a lesser detrimental effect on knee flexion than both hamstring tendon harvesting techniques. In early studies, no significant difference in extension or flexion strength of the knee following hamstring tendon harvesting for ACL reconstruction was observed [25]. However, with the advancement of more exact measurement techniques, it was discovered that there were some significant differences in knee deep-flexion torque. Reduced torque values were seen in these studies. When the torque curves were evaluated, the apex of the curve shifted left after hamstring harvest. According to these studies, the semitendinosus and gracilis muscles are essential deep flexors for knee torque [8,26]. Following this research, novel surgical procedures for protecting one of these two muscles (semitendinosus or gracilis) were described. These surgical techniques harvest only semitendinosus muscle as an autograft, preserving gracilis muscle in the process. It was determined that preserving the gracilis muscle resulted in improved knee flexion function [27,28].

Harvesting only one tendon brings new issues. To achieve sufficient graft thickness, the semitendinosus tendon must be prepared triple or quadruple, and as a result of this requirement, tendon graft length becomes an issue in this approach. To address this issue, the “all-inside technique” was developed. In the all-inside technique, femoral and tibial sockets are drilled only halfway from the inside surface of the bones in which the graft is fixed, so the length of the quadrupled tendon graft provides enough tendon-bone contact for the healing process [29]. In quadrupled semitendinosus tendon suspensory femoral-tibial button fixation (modified all-inside) technique, femoral and tibial sockets were drilled in the same manner as in suspensory femoral fixation and a tibial interference screw fixation ACLR techniques. The sockets were the same, but only the semitendinosus tendon was prepared as an autograft in a triple or quadruple manner. The autograft was then attached to the femoral socket with a suspensory device and to the tibial socket with an expanded suspensory device. This fixation technique provides adequate stability for tendon autograft [19].

In this study, the concentric strength of the hamstring and quadriceps muscles were measured using a computer-controlled isokinetic dynamometer (Humac Norm Testing and Rehabilitation System, CSMI, Stoughton, MA, USA). Patients’ healthy knees were tested as a control group. The strength of all patients’ operated and healthy knees was determined at 60/180/240° s^−1^ angular velocities. This study was designed to be consistent with past research in the orthopedic literature. This method produces more objective results that may be more comparable to those of other studies [22,25,26,29,30]. Flexion and extension strengths of the operated and non-operated knees were evaluated in both groups, and in-group and intergroup comparisons were also made between the operated and non-operative knees. Although hamstring grafts were harvested for reconstruction and the extensor mechanisms were intact, assessments revealed a significant loss of strength in knee extension at all three velocity levels in Group 1 (60°, 180°, and 240°) and two velocity levels in Group 2 (60° and 240°). This weakness was evaluated as the result of quadriceps femoris muscle atrophy, which occurred because of tourniquet application during surgery and prolonged immobilization before, during and after surgery [31]. When flexion torque strength was compared between operated groups at 60°/180°/240° s^−1^ angular velocities, no statistically significant difference was observed for flexion torque strength. According to this result, preserving the gracilis muscle does not affect the decrease in flexion torque strength. When compared to previously published research on this condition, our finding is consistent with them [25,30].

According to the results muscle strength of the non-operated knees of Group 1 was superior to the non-operated knees of Group 2. But there was no statistically significant difference between the operated knees of Groups 1 and 2. These findings strongly implied the quadrupled semitendinosus tendon suspensory femoral-tibial button fixation technique had a more detrimental effect than the classical ACLR technique. But when the measurement values were evaluated in detail, there was an intersection of confidence interval levels. As a result, these findings could be interpreted as negligible.

## 6. Limitations

While demographic data were similar between groups, muscle strength values for control (non-operative) knees were not. This undesired circumstance occurred coincidentally. To minimize the effect of this variability on statistical analyzes, covariance analyses were used to evaluate the dynamometric results. This method eliminated the possibility of obtaining erroneous statistical data. Although the number of cases seems to be statistically low, 64 cases is a reasonable number according to similar studies in the orthopedic literature.

The retrospective design and short follow-up period were other limitations of this study.

## 7. Conclusions

As a result, this study demonstrated that patients who underwent ACL reconstruction technique with quadrupled semitendinosus tendon suspensory femoral-tibial button fixation had comparable muscle strength and knee function to those who underwent ACL reconstruction with four-strand semitendinosus-gracilis tendons suspensory femoral fixation and a bioabsorbable tibial interference screw fixation. Although particular results need more interpretation, this study tried to contribute to the current orthopedic literature as a promoting paper. Even if there were no superior results for knees treated with a modified almost all-inside technique, sparing the gracilis tendon is consistent with the current minimally invasive surgery concept [32].

## Figures and Tables

**Table 1:** Comparison of the descriptive characteristics of the subjects according to the types of surgery.

Variables	Technique	N	Mean ± SD	*t*	*p*
**Age**	Group 1	32	26.15 ± 8.48	1.898	0.062
	Group 2	32	22.65 ± 6.17		
**Height (cm)**	Group 1	32	79.00 ± 10.63	0.540	0.591
	Group 2	32	77.65 ± 9.22		
**Weight (kg)**	Group 1	32	179.37 ± 6.19	−0.167	0.868
	Group 2	32	179.62 ± 5.80		
**BMI (kg/m^2^)**	Group 1	32	24.45 ± 2.23	0.695	0.490
	Group 2	32	24.05 ± 2.35		

**Table 2:** Comparison of operated and non-operated sides of the patients in Group 1 and Group 2.

Group 1	Operated Side	Non-Operated Side	*t*	*p*	ES	%95 CI	
	Mean ± SD	Mean ± SD				LB	UB
**60 Ex (Nm)**	164.78 ± 42.99	196.65 ± 38.75	−5.16	0.000	0.77	−44.32	−19.23
**180 Ex (Nm)**	109.81 ± 26.73	129.50 ± 22.01	−4.69	0.000	0.80	−28.23	−11.13
**240 Ex (Nm)**	96.53 ± 24.97	109.09 ± 22.68	−3.40	0.002	0.53	−20.08	−5.03
**60 Flx (Nm)**	114 ± 29.85	116.19 ± 25.87	−0.311	0.758	0.07	−13.24	9.74
**180 Flx (Nm)**	74.96 ± 15.51	85.62 ± 16.81	−3.28	0.003	0.65	−17.26	−4.04
**240 Flx (Nm)**	70.43 ± 19.34	79.40 ± 16.16	−3.34	0.002	0.50	−14.44	−3.49
**Group 2**	**Operated side**	**Non-Operated side**	***t***	***p***	**ES**	**%95 CI**	
	**Mean ± SD**	**Mean ± SD**				**LB**	**UB**
**60 Ex (Nm)**	146.12 ± 23.77	165.90 ± 25.49	−6.62	0.000	0.80	−25.86	−13.69
**180 Ex (Nm)**	89.53 ± 19.21	95.40 ± 19.22	−1.94	0.061	0.30	−12.04	0.29
**240 Ex (Nm)**	72.12 ± 15.79	79.87 ± 20.89	−3.153	0.004	0.41	−12.76	−2.73
**60 Flx (Nm)**	96.81 ± 18.29	99.75 ± 20.75	−1.04	0.304	0.15	−8.67	2.79
**180 Flx (Nm)**	63.34 ± 16.63	64.75 ± 16.88	−0.544	0.590	0.08	−6.67	3.86
**240 Flx (Nm)**	52.65 ± 13.03	59.19 ± 19.22	−2.581	0.015	0.39	−11.69	−1.37

Abbreviations: CI, confidence interval; ES, effect size; LB, lower bound; SD, standard deviation; UB, upper bound; Flx, flexion; Ex, extension. Note: *t*: the result of the independent-sample *t*-test; *p*: expressed as a 95% CI.

**Table 3:** Comparison of operated and non-operated sides of the patients in Group 1 and Group 2.

Variables	Group 1 Non-Operated Side	Group 2 Non-Operated Side	*t*	*p*	ES	%95 CI	
	Mean ± SD	Mean ± SD				LB	UB
**60 Ex (Nm)**	196.65 ± 38.75	165.90 ± 25.49	3.75	0.000	0.93	14.35	47.14
**180 Ex (Nm)**	129.50 ± 22.00	95.40 ± 19.22	6.60	0.000	1.65	23.76	44.41
**240 Ex (Nm)**	109.09 ± 22.68	79.87 ± 20.89	5.36	0.000	1.39	18.32	40.11
**60 Flx (Nm)**	116.18 ± 25.87	99.75 ± 20.75	2.80	0.007	0.75	4.71	28.16
**180 Flx (Nm)**	85.62 ± 16.81	64.75 ± 16.88	4.95	0.000	1.31	12.45	29.29
**240 Flx (Nm)**	79.40 ± 16.16	59.18 ± 19.22	4.55	0.000	1.13	4.43	29.09

Abbreviations: CI, confidence interval; ES, effect size; LB, lower bound; SD, standard deviation; UB, upper bound; Flx, flexion; Ex, extension. Note: *t*: the result of the independent-sample *t*-test; *p*: expressed as a 95% CI.

**Table 4:** Comparison of operated sides of the patients in Group 1 and Group 2 with covariance analysis.

Variables	Group 1 Operated Side	Group 2 Operated Side	*t*	*p*
	Mean ± SD	Mean ± SD		
**60 Ex (Nm)**	164.87 ± 42.99	146.12 ± 23.77	0.004	0.947
**180 Ex (Nm)**	109.81 ± 26.73	89.53 ± 19.21	0.070	0.792
**240 Ex (Nm)**	96.53 ± 24.97	72.12 ± 15.79	0.077	0.783
**60 Flx (Nm)**	114.43 ± 29.85	96.03 ± 18.33	1.178	0.282
**180 Flx (Nm)**	74.96 ± 15.51	63.34 ± 16.63	1.923	0.171
**240 Flx (Nm)**	70.43 ± 19.34	52.65 ± 13.03	1.569	0.215

## Data Availability

The data is available and if needed corresponding author can share.
